# Treatment Adherence and Health Outcomes in MSM with HIV/AIDS: Patients Enrolled in “One-Stop” and Standard Care Clinics in Wuhan China

**DOI:** 10.1371/journal.pone.0113736

**Published:** 2014-12-01

**Authors:** Wang Zhou, Min Zhao, Xia Wang, Robert F. Schilling, Sheng Zhou, Hong-Yan Qiu, Nian-Hua Xie, Man-Qing Liu, Han-Sheng Dong, Zhong-Zhao Yao, Thomas Cai

**Affiliations:** 1 Wuhan Centers for Disease Control and Prevention, Wuhan, Hubei, China; 2 Wuhan Institute of Dermatovenereology, Wuhan, Hubei, China; 3 Department of Social Welfare, Luskin School of Public Affairs, University of California Los Angeles, Los Angeles, California, United States of America; 4 Department of Epidemiology, Bloomberg School of Public Health, Johns Hopkins University, Baltimore, Maryland, United States of America; 5 AIDS Care of China, Nanning, Guangxi, China; UCSF, United States of America

## Abstract

**Background:**

Conducted in Wuhan China, this study examined follow-up and health markers in HIV patients receiving care in two treatment settings. Participants, all men who have sex with men, were followed for18–24 months.

**Method:**

Patients in a “one-stop” service (ACC; N = 89) vs those in standard care clinics (CDC; N = 243) were compared on HIV treatment and retention in care outcomes.

**Results:**

Among patients with CD4 cell count ≦350 cells/µL, the proportion receiving cART did not differ across clinic groups. The ACC was favored across five other indicators: proportion receiving tests for CD4 cell count at the six-month interval (98.2% vs. 79.4%, 95% CI 13.3–24.3, *p* = 0.000), proportion with HIV suppression for patients receiving cART for 6 months (86.5% vs. 57.1%, 95% CI 14.1–44.7, *p* = 0.000), proportion with CD4 cell recovery for patients receiving cART for 12 months (55.8% vs. 22.2%, 95% CI 18.5–48.6, *p* = 0.000), median time from HIV confirmation to first test for CD4 cell count (7 days, 95% CI 4–8 vs. 10 days, 95% CI 9–12, log-rank *p* = 0.000) and median time from first CD4 cell count ≦350 cells/µL to cART initiation (26 days, 95% CI 16–37 vs. 41.5 days, 95% CI 35–46, log-rank *p* = 0.031). Clinic groups did not differ on any biomedical indicator at baseline, and no baseline biomedical or demographic variables remained significant in the multivariate analysis. Nonetheless, post-hoc analyses suggest the possibility of self-selection bias.

**Conclusions:**

Study findings lend preliminary support to a one-stop patient-centered care model that may be useful across various HIV care settings.

## Introduction

HIV/AIDS is found in all regions of China [1], and at the end of 2011, an estimated 780,000 people were living with HIV infection [2]. Men who have sex with men (MSM) accounted for 29% of new cases, and 17% of persons living with AIDS [2]–[5]. Combination antiretroviral therapy (cART) can reduce HIV morbidity, mortality and transmission [6]–[9], but efficacy requires access to treatment and high levels of adherence [10], [11]. A “cascade effect” has been observed, in which not everyone with HIV infection is diagnosed, not all of those who are diagnosed are in care, not all of those in care are receiving antiretroviral therapy, and viral suppression is incomplete among some who are on such therapy [12].

Chinese investigators [13] evaluated all 83,556 patients 16 years and older who tested HIV positive from 2005 to 2009 in Yunnan and Guangxi provinces. Slightly more than a third (37%) received a CD4 cell count within 6 months of receiving a diagnosis of HIV; the rate of CD4 cell count testing was still only 62% by 2009. These and many other studies indicate that many HIV infected individuals are receiving care that is far from optimal, even after they are diagnosed.

Improving the clinical care experience could help patients to achieve higher levels of participation and retention in health care and in cART protocols in particular, leading to prolonged survival [14], [15]. A recent Cochrane review [16] summarized 16 studies of decentralized antiretroviral therapy service delivery for HIV patients in lower and middle income countries. The authors concluded that attrition appears to be lower in partially and fully decentralized models of treatment. A systematic review from the United States [17] found that the best evidence favored strengths-based case management—encouraging clients to recognize their own abilities to access resources and solve problems. Other evidence-based strategies included reducing structural- and system-level barriers, inclusion of peers as part of a health care team and using community-based organizations to engage HIV-infected persons. Collaborators at four evaluation sites adapted a patient navigation model first developed for cancer care and sought to assess its effectiveness with 400 HIV-infected individuals—predominantly non-Caucasian, poorly educated males who did not live in their own homes [18]. A key program element was the use of non-clinician assistants to help patients to access healthcare resources. Longitudinal patient interviews and medical record reviews indicated that the patient navigation intervention significantly reduced all barriers to care at 6-month and 12-month follow-up. Increased retention was associated with undetectable viral loads and increased medical visits.

Emphasizing the commonalities between HIV management and other chronic disease care, other observers have called for a self-management model with patients assuming an active and informed role in healthcare decision making [19]. Their review of the literature outlined 14 elements in three broad categories of physical health, psychological functioning, and social relationships. Selected common elements included treatment adherence, self-monitoring of physical status, and collaborative relationships with healthcare providers. Mayer and colleagues [20] observed that MSM have unique health-care needs, including an increased susceptibility to infection with HIV and sexually transmitted infections, but also internalized stigma that is associated with depression, anxiety and substance use. Finally, a 2011 review of the elements of a “comprehensive surveillance system of HIV care” found that “Data on HIV care programmes in developing countries are generally fragmented and weak, focusing primarily on outcomes of patients on ART.”[21] The authors argued for “HIV care that integrates multiple elements…to provide evidence-based data to optimize quality of care and improve survival.”

The literature on “one stop”/integrated HIV care offers useful program guidelines, but few studies have formally evaluated these models of care. The present study in central China examined patient outcomes in a new “one-stop” care program and in existing clinics.

Under China's “Four Free and One Care” policies [22], [23], local facilities of the Centers for Disease Control and Prevention (CDC) deliver case identification, follow-up, and ongoing care. In June, 2010, WHO and UNAIDS advanced the Treatment 2.0 initiative [5], [24]. The most important recommendations were decentralizing high-quality services to facilitate early diagnosis and retention in care; streamlining and coordinating system structures and pathways to reduce user burden and time; closer service collaboration, including harm reduction, attending to other sexually transmitted infections; perhaps through one-stop clinics offering multiple interventions by the same treatment team and coordination with noncommunicable disease programs [5].

The National CDC subsequently outlined a “Treatment 2.0” plan for China, and the Wuhan CDC was the first site to initiate a pilot project integrating a range of public health resources within a “one-stop” service delivery protocol for individuals with HIV/AIDS infection. The intent of the present comparison study is to describe the treatment experience, follow-up participation and disease progression among patients who chose to enroll in either one of two delivery models of HIV/AIDS care.

## Materials and Methods

### Background and setting

The CDC-initiated Wuhan AIDS Care Center (ACC) is a comprehensive “one-stop” program for people living with HIV (PLHIV). The pilot effort was initiated at a single clinic, permitting a comparison of the demonstration project and standard care provided in the existing CDC system. The new clinic included counseling, testing, diagnosis, treatment and health education provided by physicians, nurses and workers from community-based organizations (CBOs) serving MSM. The “one-stop” services included cART provision, regular follow-up, referral for Tuberculosis (TB) screening and treatment, referral for AIDS-related or non-AIDS-related diseases, risk behavior prevention, and psychological support. The ACC maintained close linkages with district CDCs, the Tuberculosis Institute, comprehensive general hospitals, and the designated AIDS hospital (an infectious disease hospital, usually one per city or region, receiving patients with AIDS-related medical issues, such as serious opportunistic infections and tumors). The ACC provided as many services as possible within the facility; when additional services, such as diagnosis and treatment of TB, were needed, ACC staff actively linked the patient to other elements within the CDC system. For example, ACC staff often telephoned collaborating health care providers in the presence of the patient and routinely followed up with patients to be sure that the referral was completed and to inquire about patient's understanding of the test, procedure or consultation. The ACC trained members of CBOs on knowledge and skills of HIV prevention and provides AIDS/-related materials and condoms. The CBOs coordinated closely with the ACC, and assigned staff to the ACC clinic to work directly with patients. CBO staff provided a range of services including referral, transportation, information and emotional support. As stigma is inversely associated with HIV testing and treatment adherence [25], CBO staff discussed issues of stigma, homophobia and discrimination informally and in discussion groups with patients. MSM peer educators served as positive role models, addressed patients' concerns about potential discrimination by health care providers, and advocated for patients when necessary.

Patients in the usual-care CDC clinic could potentially access the same kinds of services available to patients in the ACC clinic; however, some districts provide a range of services within the primary care clinic or in nearby facilities, while others offer only basic care. The comprehensiveness of care depends on the degree to which the primary health care provider is aware of what is available and the degree to which effective linkages are made. All CDC clinics included counseling, testing, health education, cART provision, and regular follow-up.

All 13 CDC districts/clinics were involved in the study; of these, only two provide extensive services within the primary care clinic, while others offer limited care, such as blood pressure, blood sugar and routine assays of blood and urine etc. In all districts in Wuhan, specific tests (e.g., ALT, BUN, X-ray, CD4 cell count and HIV-RNA test) are conducted in a designated AIDS hospital with expertise in HIV and other contagious diseases.

The major ACC vs CDC distinctions were the service providers and the service delivery arrangements. The CDC clinics only have public health workers; the ACC has physicians, nurses and workers from CBOs. Some CBOs refer patients to CDC units, but CBO staff do not work at CDC clinics. CDC patients did not receive peer education, psychological support, or other services provided by CBO staff. For CDC patients, the relevant clinical assays were conducted upon referral to nearby facilities; e.g., tests for HIV-RNA and CD4 were carried out after referral to Wuhan CDC. cART-related side effects and other clinical conditions were referred to the designated AIDS hospital or comprehensive hospitals; whereas, for ACC patients, all of these procedures and protocols, except TB screening, were performed in the ACC setting.

### Design and procedure

**Figure 1:**
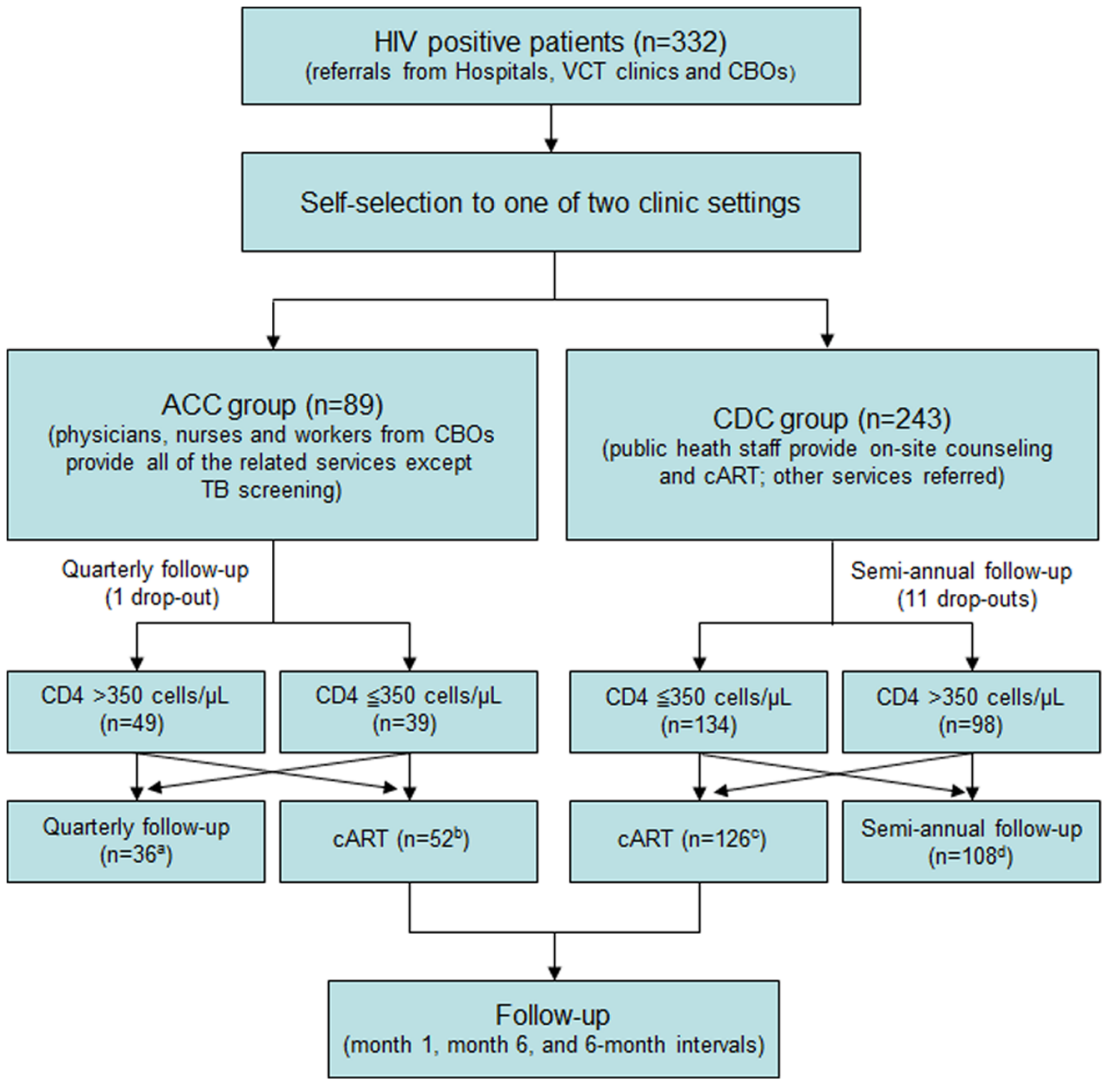
Enrollment and follow-up. (Note: a. includes 3 patients whose CD4 cell count is ≦350 cells/µL; b. includes 16 patients whose CD4 cell count is >350 cells/µL; c. includes 2 patients whose CD4 cell count is >350 cells/µL; d. includes 10 patients whose CD4 cell count is ≦350 cells/µL.)

The enrollment period spanned July 1, 2011 to June 30, 2012; the final follow-up assessment was December 31, 2013. Figure 1 displays the enrollment and the follow-up of patients. Observed patients were 332 men who have sex with men, with HIV infection confirmed between July, 2011 and June, 2012, and indicating a desire to receive follow-up and treatment. As outlined in the informed consent protocol, patients could choose either the newly established ACC (89 patients) or the existing CDC system of care (243 patients). ACC participants paid a fee of approximately 200 Yuan (approx. $32) per month, although all patients had insurance that reimbursed 80% of this cost. Most patients in the ACC (75%) and the CDC (56%) were referred by CBOs. Others were diagnosed in various general hospitals or referred by voluntary counseling and testing (VCT) clinics.

Demographic and biomedical data for all patients were recorded at baseline and relevant biomedical data were obtained at follow-up interviews. ACC patients were followed on a quarterly basis and the usual-care CDC patients were followed on a semi-annual schedule.

Each follow-up visit included: a physical examination for basic health conditions including breath frequency, heart rate, blood pressure and body weight; indicated screening for sexually transmitted infections and tuberculosis; routine assays of blood and urine (syphilis, red blood cell count and hemoglobin, white blood cell count and sort, urine color and turbidity, pH, glucose, bilirubin, protein, occult blood, ketone, nitrite and leukocytes esterase), tests for alanine aminotransferase (ALT), blood urea nitrogen (BUN) and CD4 cell count. ACC and CDC patients received respectively two and one HIV-RNA tests annually. In both the ACC and CDC clinics, staff sought to begin CD4 cell counts soon after a confirmatory HIV test and to begin cART immediately following a cell count ≦350 cells/µL. cART was delayed for a few patients (2, ACC; 5, CDC), when immediate initiation of antiretroviral treatment was contraindicated.

In the follow-up assessments, date-anchored CD4 counts, HIV-RNA tests and cART treatments were used to determine health status across six health care indicators (HCIs), four relevant to HIV treatment and patient adherence, and two measuring recognized markers of disease status [26]. These values were used to compute: 1) the proportion receiving tests for CD4 cell count at the six-month interval; 2) the proportion receiving cART in patients with CD4 count ≦350 cells/µL; 3) the proportion with HIV suppression (viral load [VL] ≦50 copies/mL) for patients receiving cART for 6 months [27]; 4) the proportion with CD4 cell recovery (CD4 cell increase ≧150 cells/µL) for patients receiving cART for 12 months [27]; 5) time from HIV confirmation to the first test for CD4 cell count; 6) time from the first CD4 cell count ≦350 cells/µL to cART initiation.

The Institutional Review Board of Wuhan CDC approved this study, and written informed consents were obtained from all the patients.

### Statistical analyses

Data were analyzed with the software SAS version 9.13 (SAS Institute, Cary, NC, USA). Categorical data were compared with the chi-square (χ^2^) test and Fishers exact probability test or the Mann-Whitney test. If abnormal distributions were found, the Wilcoxon rank sum test was used. Median times to test for CD4 cell count and cART initiation were compared through Kaplan-Meier curves and log rank tests. Cox proportional hazards regression was used to identify factors influencing adherence. Statistical tests were two-sided with alpha  = 0.05.

## Results

### Socio-demographic and health-related descriptors

As of December 2013, the mean observation times for the ACC clinic and the CDC clinic were 2.02 person-years (interquartile range [IQR] 1.78–2.21 person-years) and 1.94 person-years (IQR 1.64–2.21). ACC patients ranged in age from 18–68, with an average age of 32.7 (95% confidence interval [CI] 30.6–34.8). CDC patients ranged in age from 16–82, and their average age was 34.7 (95% CI 32.9–36.3). Many participants had a junior college or higher degree, but the education of ACC patients was higher than that of CDC patients (*p* = 0.012). More ACC patients had temporary or permanent jobs, whereas larger proportions of the CDC group were unemployed or students (*p* = 0.000). At baseline, the two groups did not differ significantly on any of ten clinical tests. Approximately two-thirds of patients reported having never been married (Table 1).

**Table 1:** Socio-demographic characteristics and baseline health-related conditions, by clinic.

Category (variable)	Number (%) or value (range), ACC clinic (N = 89)	Number (%) or value (range), CDC clinic (N = 243)	Test*	*p* value
**Patient referral source**				
CBOs	67 (75.3)	136 (56)	10.246	0.006
Hospitals	16 (18)	76 (31.3)		
VCT clinics	6 (6.7)	31 (12.7)		
**Median age (y)**	30.5 (18–68)	31 (16–82)	−0.274	0.784
**Marriage**				
Unmarried	61 (68.5)	164 (67.5)	1.780	0.411
Married	12 (13.5)	23 (9.5)		
Divorced	16 (18)	56 (23)		
**Education**				
Junior high school or lower	9 (10.1)	61 (25.1)	8.798	0.012
Senior high school	29 (32.6)	66 (27.2)		
Junior college or higher	51 (57.3)	116 (47.7)		
**Employment**			47.953	0.000
Server	43 (48.3)	41 (16.9)		
Unemployed	11 (12.4)	69 (28.4)		
Staff	11 (12.4)	15 (6.2)		
Worker	10 (11.2)	20 (8.2)		
Student	6 (6.7)	42(17.3)		
Retired	2 (2.4)	12 (4.9)		
Other	6 (6.7)	44 (18.1)		
**Health-related conditions**				
Median body weight (Kg)	64.3 (47.1–87.9)	62.5 (41–100)	−1.601	0.109
Abnormal white blood cell count†	23 (25.8)	64 (26.3)	0.008	0.928
Abnormal hemoglobin†	9 (10.1)	28 (11.5)	0.131	0.718
Abnormal ALT†	7 (7.9)	17 (7)	0.073	0.786
Abnormal BUN†	16 (18)	43 (17.7)	0.004	0.953
Median CD4 cell count (cells/µL)	391 (39–1,017)	345 (5–1,056)	−1.472	0.141
Median VL (copies/mL)	19,000 (1,300–999,999)	24,000 (20–999,999)	−0.571	0.568
HbsAg positive	12 (13.5)	30 (12.3)	0.076	0.782
Anti-HCV positive	2 (2.2)	5 (2.1)	0.011	0.915
Tuberculosis	9 (10.1)	22 (9.1)	0.086	0.769

*The Chi-square (χ^2^) test was used for proportions and the Mann-Whitney test was used for medians.

† The normal ranges for white blood cell count, hemoglobin, ALT and BUN are 4.10×10^9^ cell/L, 120–160 g/L, 0–40 IU/L and 3.2–6.0 mmol/L, respectively.

### HIV treatment and health care outcomes

In the ACC clinic, one patient missed all follow-up occasions. Of the remaining 88, 44.3% (39/88) had CD4 cell counts of ≦350 cells/µL, 59.1% (52/88) received cART treatment, including 36 patients with CD4 cell count ≦350 cells/µL and 16 with CD4 cell count>350 cells/µL; 90.4% (47/52) were followed for 18–24 months. A total of 243 CDC patients were initially observed, but 11(4.5%) moved away or provided inaccurate contact information. Of the remaining 232 patients, 54.3% (126/232) received cART treatment, including 124 patients with CD4 cell count ≦350 cells/µL and two patients with CD4 cell count>350 cells/µL; 67.5% (85/126) were followed for 18–24 months.

Nine CDC patients died, a mortality rate of 3.97 per 100 person-years (95% CI 1.50–6.44). Their median age was 46 (39–82); their median CD4 cell count at baseline was 204 (5–791) cells/µL; the mean time between HIV confirmation and death was 38 (1–481) days; six died prior to initiation of cART. Causes of death were AIDS-related opportunistic infections (6), suicide (2) and acute myocardial infarction (1). No ACC patients died. Baseline health indicators of drop-out and deceased cases within the CDC clinic are shown in Table 2. In comparison with those followed, drop-out cases were significantly younger and had higher median CD4 cell counts at baseline. Deceased patients were significantly older and, at baseline, had lower median CD4 cell counts than surviving patients.

**Table 2:** Baseline health-related conditions in CDC clinic patients: Retained vs dropout and living vs deceased.

Category (variable)	Follow-up vs Drop-out, number (%) or value (range)			Living vs Deceased, number (%) or value (range)		
	Follow-up (n = 232)	Drop-out (n = 11)	*p value*	Living (n = 223)	Deceased (n = 9)	*p value*
Median age (y)	31.5 (16–82)	25 (21–49)	0.023	30 (16–82)	46 (39–82)	0.018
Median body weight (Kg)	61.5 (41–100)	65.5 (54.5–79)	0.105	62 (41–100)	63.5 (49–73)	0.102
Abnormal white blood cell count*	62 (26.7)	2 (18.2)	0.530	58 (26)	4 (44.4)	0.220
Abnormal hemoglobin*	27 (11.6)	1 (9.1)	0.796	23 (10.3)	4 (44.4)	0.002
Abnormal ALT*	17 (7.3)	0	–	16 (7.2)	1 (11.1)	0.657
Abnormal BUN*	43 (18.5)	0	–	42 (18.8)	1 (11.1)	0.559
Median CD4 cell count (cells/µL)	345 (5–1,056)	560 (382–1024)	0.015	345 (5–1,056)	204 (5–791)	0.032
Median VL (copies/mL)	24,000 (20–999,999)	16,000 (2,400–999,999)	0.097	24,000 (20–999,999)	36,000 (2,400–999,999)	0.089
HBsAg positive	29 (12.5)	1 (9.1)	0.737	27 (12.1)	2 (22.2)	0.368
Anti-HCV positive	5 (2.2)	0	–	5 (2.2)	0	–
Tuberculosis	22 (9.5)	0	–	19 (8.5)	3 (33.3)	0.013
Receiving cART	126 (54.3)	0	–	123 (55.2)	3 (33.3)	0.198

*The normal ranges for white blood cell count, hemoglobin, ALT and BUN are 4–10×10^9^ cell/L, 120–160 g/L, 0–40 IU/L and 3.2–6.0 mmol/L, respectively.

**Figure 2:**
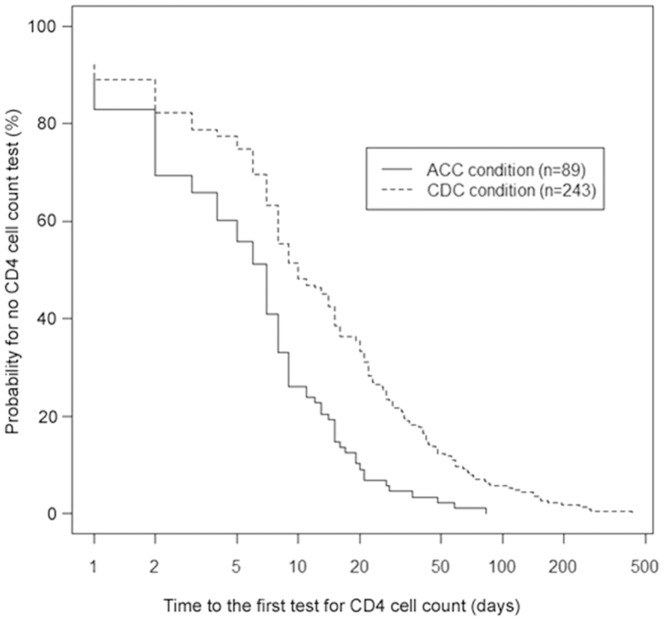
Time from HIV confirmation to the first test for CD4 cell count (HCI 5) for the ACC clinic (median: 7 days, 95% CI 5–8) and CDC clinic (median: 10 days, 95% CI 8–14) (log-rank χ^2^ = 26.178, *p* = 0.000).

**Figure 3:**
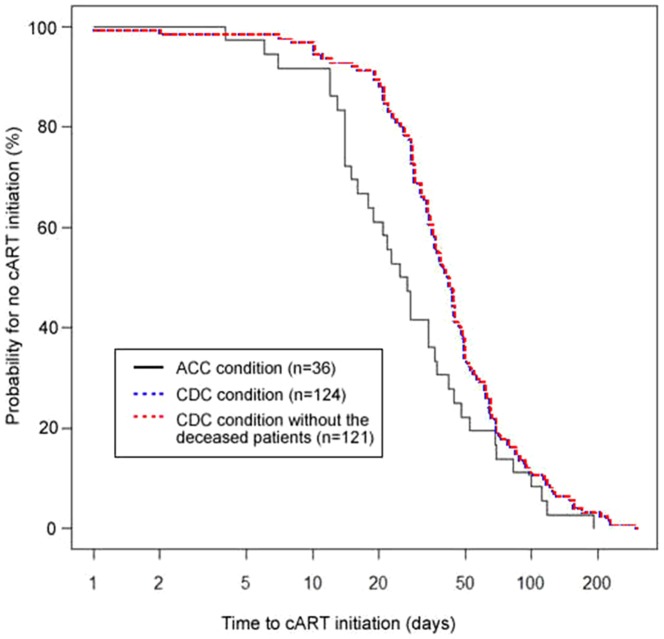
Median time from first CD4 cell count ≦350 cells/µL to initiation of cART (HCI 6) for ACC clinic (median: 26 days, 95% CI 18–37) and the CDC clinic (median: 41.5 days, 95% CI 35–46) (log-rank χ^2^ = 4.632, *p* = 0.031).

As presented in Table 3 and figures 2–3, patients' follow-up adherence was measured with six health care indicators (HCIs) relevant to CD4 cell counts, HIV-RNA tests and cART treatment. Outcomes significantly favored the ACC patients over the CDC patients on every indicator except HCI 2 (the proportion receiving cART in patients with CD4 count ≦350 cells/µL). Figure 2 displays the Kaplan-Meier survival curve for time from HIV confirmation to the first test for CD4 cell count (HCI 5). Figure 3, depicting time from the first CD4 cell count ≦350 cells/µL to cART initiation (HCI 6), includes a third survival curve for the CDC group with the deceased patients removed–virtually identical to the curve for the entire CDC group.

**Table 3:** ACC vs CDC patients on treatment outcomes.

Health care indicator (HCI)	Proportion (95% CI), ACC clinic	Proportion (95% CI), CDC clinic	Difference (95% CI)	*p* value
**HCI 1**: The proportion (%) receiving tests for CD4 cell count at the six-month interval	98.2 (222/226) (96.5–99.9)	79.4 (475/598*) (76.2–82.7)	18.8 (13.3–24.3)	0.000
**HCI 2**: The proportion (%) receiving cART in patients with CD4 cell count ≦350 cells/µL	92.3 (36/39) (83.9, 100)	93.2 (133/143) (88.8, 97.2)	−0.9(−10.0, 8.2)	0.842
**HCI 3**: The proportion (%) with HIV suppression (VL ≦50 copies/mL) for patients receiving cART for 6 months	86.5 (45/52) (77.3–95.8)	57.1 (72/126) (48.5–65.8)	29.4 (14.1–44.7)	0.000
**HCI 4**: The proportion (%) with CD4 cell recovery (CD4 cell increase ≧150 cells/µL) for patients receiving cART for 12 months	55.8 (29/52) (42.3–69.3)	22.2 (28/126) (15.0–29.5)	33.5 (18.5–48.6)	0.000

*Six patients who died within the initial 6-month follow-up period were not included in the analysis.

### Factors associated with adherence

Cox proportional hazards regression was used to identify predictors of HCI 1, HCI 3 and HCI 4. For HCI 1, independent variables included clinic setting and four socio-demographic variables listed in table1. For HCI 3 and HCI 4, independent variables included clinic setting, four socio-demographic variables, ten health-related conditions listed in Table 1 (body weight was stratified into ≦60 Kg and >60 Kg; baseline CD4 cell count was stratified into ≦350 cells/µL and >350 cells/µL; baseline VL was stratified into ≦100,000 copies/mL and >100,000 copies/mL; other health-related conditions were grouped as normal/abnormal), the first CD4 cell count (stratified into ≦200 cells/µL and >200 cells/µL) and time from the HIV confirmation to the first CD4 cell count (stratified into ≦10 days and >10 days), time from the first CD4 cell count ≦350 cells/µL to cART initiation (stratified into ≦30 days and >30 days). When all of these independent variables were examined in the model, no variables for HCI 1 remained in the Cox proportional hazards regression equation. The significant predictors for HCI 3 and HCI 4 are summarized in Table 4. A multivariate analysis employing a Cox proportional hazards regression model found that the variables related to HCI 3 were clinic setting (estimated RR: 0.131; 95% CI: 0.083–0.204; *p* = 0.000). The variables related to HCI 4 were clinic setting (estimated RR: 0.130; 95% CI: 0.650–0.259; *p* = 0.000), baseline ALT value (estimated RR: 1.831; 95% CI: 1.041–3.219; *p* = 0.036), baseline CD4 cell count (estimated RR: 3.015; 95% CI: 1.456–6.244; *p* = 0.003).

**Table 4:** Multivariate Cox proportional hazards model for health care indicators (HCIs).

Health care indicator (HCI)	Variables	B	Sb	χ^2^	*p* value	RR	95% CI
**HCI 3**	Clinic setting	−2.036	0.228	79.421	0.000	0.131	0.083–0.204
**HCI 4**	Clinic setting	−2.043	0.352	33.651	0.000	0.130	0.065–0.259
	Baseline ALT value	0.605	0.288	4.409	0.036	1.831	1.041–3.219
	Baseline CD4 cell count	1.104	0.371	8.824	0.003	3.015	1.456–6.244

## Discussion

Consistent medical follow-up is essential for measuring patients' disease progression, monitoring treatment effects and providing health education and behavioral intervention [28], [29]. Although many reports have focused on treatment adherence among PLHIV, fewer studies have described follow-up, adherence and health outcomes of patients enrolled in various care models [30]–[32]. The measurement scheme of this comparative observational study was superior to self-report employed in many previous adherence studies [20], [33]. The patient groups did not differ on any biomedical indicator at baseline. The ACC patients were more likely to be employed, but no demographic variables remained significant in the multivariate analysis.

Treatment outcome determination cannot entirely disentangle measurement follow-up from the monitoring components of the intervention itself [34]. Some study results could be attributable to the synergy between the one-stop program and frequent follow-up of the ACC patients in the initial period of the study. ACC patients were initially followed quarterly, vs semi-annually for the CDC patients, but the follow-up protocols for both groups were the same after initiation of cART [27]. Differential follow-up frequency between the ACC and CDC patients before initiation of ART could influence between-group differences on HCI 1 and HCI 5, but would not likely influence other outcome variables.

The substantial differences in mortality between the ACC (0) and CDC (9) groups were unexpected. Six of nine deaths occurred within six months of enrollment. Compared to the CDC patients who lived, the deceased patients were substantially older, and, at baseline, had lower CD4 cell counts and were more likely to have abnormal hemoglobin values (Table 2). Because all of these medically compromised cases were in the CDC clinic, any imputation for missing cases would have resulted in larger between-group scores than those provided in Table 3. Overall attrition was 3.8%, comparing favorably to the 30% reported in a somewhat related review of ART adherence studies [35]. Eleven of 12 missing cases were in the CDC group, a between-group differential higher than the 9% estimated in the same review. Among the CDC patients, compared to the retained cases, individuals lost to follow-up were younger, had significantly higher CD4 cell counts at baseline, and appeared to be healthier on five of eight other disease parameters. Attrition is never desirable in any follow-up study, but it this instance any resulting bias is likely to be in the direction of smaller between-group outcomes. Plausibly, these younger, relatively healthy men were less concerned about their diagnosis of HIV infection. If confirmed in other studies [36], [37], this observation would point toward allocation of resources for educating and supporting newly diagnosed HIV/AIDS patients of younger age with relatively favorable health profiles. Such efforts merit consideration because young, sexually active men who have sex with men continue to show high rates of risk behavior and HIV infection across multiple regions.

No cases are missing for indicator 5. For indicator 1, data are missing for the six individuals who died within the initial 6-month follow-up period. The between-group result was virtually the same when missing cases were replaced and imputed a positive value for “complete CD4 count at six months” (98.9% [88/89] vs. 87.2% [212/243], *p* = 0.001). Missing data would have little effect on HCIs 2, 3, 4 and 6, as these outcomes were assessed only for those participants whose CD4 counts were almost always less than 350 cells/µL–while all drop-outs had high CD4 counts. It appears unlikely that the loss of cases biased study results.

As noted above, interpretations of a non-randomized study must always include the possibility that unmeasured variables account for observed between-group differences. Another important study limitation is the potential heterogeneity in the quality of HIV care within the CDC and ACC clinics. The CDC clinics do not have physicians, only public health workers who distribute cART drugs to patients under the guidance of physicians and other staff with substantial clinical training. Although the ACC was designed as an integrated care unit, it is also possible that the structure and focus of the clinic improves the expertise of the health care providers in any number of ways that were not measured in the present study. The more favorable outcomes among patients in the ACC could be due at least in part to staffing with more highly trained personnel, and a milieu of excellence–not only the integration of care. It should be noted, however, that all CDC staff had been trained for cART provision, follow-up and basic care since 2004, and were regularly monitored by Wuhan CDC. Likewise, the ACC protocols were carefully specified, but perhaps not with the degree of precision found in a randomized clinical trial. For example, the ACC protocols did not prescribe or track the elements of the ACC partnership with CBOs, nor assess how these supportive services may have addressed stigma, homophobia, and discrimination faced by MSM, which, in turn may have favorably interacted with targeted clinical outcomes.

In sum, study findings favored the one-stop care model over the existing service model on five of six indicators, including time from initial HIV test to initial CD4 cell count, proportion of patients receiving CD4 cell count at six-months, time between the first indication of a CD4 cell count ≦350 cells/µL and the initiation of cART, CD4 cell recovery and viral suppression. Follow-up outcomes for the CDC patients, and the ACC patients in particular, compare favorably to the markers cited in the studies discussed above on follow-up participation, viral load and CD4 count.

Only 27% of patients chose the ACC clinic; as noted, the reimbursable fee may have been a disincentive. Patients who were referred by the hospital may have believed that the CDC—already known to them–could protect their confidentiality better than the unknown ACC. Notwithstanding the clinical similarity of the two clinic groups at baseline, it is possible that men who were feeling relatively well and had a positive attitude about their future were more likely to be attracted to the ACC clinic.

Interpretations of the unexpected differential mortality between groups are problematic, given the limitations of a non-randomized study. The profile of the nine deceased CDC patients is quite distinct from that of the overall study group, and it is unknown whether these patients might have lived longer had they enrolled in the ACC clinic. Very sick patients nearing death may not have wished to enroll in an unknown clinic.

Incorporating many of the recommendations in the WHO “Treatment 2.0” initiative of 2010, the ACC clinic described herein was the first site in China to initiate a pilot project specifically designed to substantially improve health care services for people living with HIV infection. The program included a comprehensive array of services including testing and diagnosis, treatment and monitoring, counseling and health education, provided by physicians, nurses and workers from community-based organizations serving MSM. The “one-stop” program was favored on three of four process indicators of care, and on indicators of CD4 cell recovery and viral suppression. Albeit compromised by possible self-selection bias, study findings are consistent with existing recommendations concerning the feasibility of the “one- stop” patient-centered approach to care of persons with HIV/AIDS.

Future studies, including designs employing randomization, could isolate specific program elements accounting for favorable patient outcomes. Also needed are replication efforts across other areas and settings, particularly those measuring the effectiveness, benefits and costs of various program designs within an overall framework of patient-centered care. Although strong claims of “effectiveness” are not possible in a comparison across self-selected groups, results from this study lend further support to a “one-stop” approach to health care for persons with HIV disease.
